# Functional Outcomes of Tibia Fractures Treated With Intramedullary Interlocking Nails by Suprapatellar Approach: A Prospective Study

**DOI:** 10.7759/cureus.40485

**Published:** 2023-06-15

**Authors:** Shree Sagar B V, Santosh S Nandi, Shreepad R Kulkarni, Rajkumar Bagewadi

**Affiliations:** 1 Orthopaedics, Shri B. M. Patil Medical College, Hospital and Research Centre, Bijapur Lingayat District Educational (BLDE) University, Vijayapura, IND

**Keywords:** functional outcome, semiextended knee, intramedullary nailing, suprapatellar approach, tibia fracture

## Abstract

Background

Tibia fractures are the most common lower extremity fractures. The subcutaneous anatomy of this long bone predisposes it to high fracture frequency in a high-energy trauma. The tibia is a major weight-bearing, long tubular bone that is axially and rotationally unstable when fractured, which ideally necessitates its surgical fixation in adults. Tibia fractures can be treated with a variety of choice of implants and surgical approaches. This study aims to assess the clinical and functional outcomes of a tibia fracture treated with intramedullary interlocking nails by a suprapatellar approach.

Methodology

A total of 32 patients were selected from patients admitted at Shri B. M. Patil Medical College and Research Centre with tibia fractures between January 2021 and May 2022. All the patients were treated with closed reduction and internal fixation with intramedullary interlocking nails by suprapatellar approach with a semi-extended knee position. All patients were followed up clinically and radiologically at regular intervals of six weeks, three months, six months, and one year. All functional outcomes were assessed based on modified Lysholm knee scores.

Results

A total of 31 patients showed union at the fracture site. One patient had nonunion and implant failure at the distal locking site, and two patients had persistent anterior knee pain at the end of one year. Functional outcome assessment based on modified Lysholm scores had excellent results, with a mean score of 95. Patients were followed up for a mean of 11.5 months. The mean time of union was observed as 12.5 months.

Conclusions

Suprapatellar tibia nailing is an effective alternative approach with ease of reduction and decreased intraoperative fluoroscopy time. The entry is in line with the medullary cavity preventing malreduction of proximal and distal tibia fractures. The additional proximal locking option also increases the stability of implant fixation.

## Introduction

Tibia fractures are one of the most common fractures of long bones, constituting about 2% of adult fractures [1,2]. Tibia shaft fractures occur with an incidence of 16.9/100,000 per year [3]. The incidence of tibial fracture has a bimodal peak at ages 20 and 50 [4].

The treatment of tibia fractures has seen the development from casting and functional bracing to intramedullary nailing and plating, as developed by Kuntscher in the 1940s [5]. Intramedullary interlocking nails are now the gold standard modality of treatment for closed tibia fractures. With varied fracture patterns based on anatomical location or varied possible complications, new advancements have been made in the concept of tibia nailing.

Intramedullary interlocking nail fixation with a semi-extended knee position has been used for proximal and distal one-third tibia shaft fractures, as described by Tornetta et al. [6,7]. A modification of the semi-extended knee technique for nailing was the suprapatellar approach by Cole [8]. The main concern with this technique is the risk of injury to the patellofemoral articular cartilage, which could cause early patellofemoral arthritis and anterior knee discomfort following intramedullary nail fixation. The risk of chondral damage can be reduced with the use of a protective sleeve.

Suprapatellar tibia nailing in semi-extended knee positions has been gaining popularity in intramedullary nailing, with the semi-extended position of nailing facilitating easy manipulation and reduction. This technique allows easier use of fluoroscopy intraoperatively, with shorter fluoroscopy exposure time [9-11]. Nail entry through the suprapatellar approach creates a parallel plane of entry in line with the sagittal axis of the tibia, facilitating ease of reduction and nail entry. This technique has a lower potential for postoperative malalignment in terms of proximal and distal tibia fractures.

## Materials and methods

We conducted a study on 32 patients with proximal and distal tibia shaft fractures, treated with suprapatellar tibia nailing at Shri B. M. Patil Medical College. Our period of study was from January 2021 to May 2022. All patients were treated with suprapatellar intramedullary interlocking nails as the definitive treatment. All patients were initially immobilized in above-knee splints, and pain management was started with intravenous analgesics. All grade 1 Gustilo Anderson fractures were given a thorough wash on presenting to the hospital and were started on prophylactic antibiotics. Preliminary clinical and laboratory assessment was done to determine the cardiopulmonary status of the patient, and operative procedures were planned accordingly. Preoperative radiographs of ipsilateral knees were also taken to consider patellofemoral space as a prerequisite for suprapatellar nailing. Preoperative nail length was measured for all patients. All patients were informed about the procedure in their languages, and both written and informed consent was obtained.

All patients were started on static quadriceps and ankle foot exercises on day one of the postoperative periods. The first sterile dressing was done on day two of the postoperative period, and bedside dynamic quadriceps exercises were started with a full range of motion allowed as tolerated. Patients were allowed for partial weight-bearing at six weeks with concerning fracture pattern and callus formation. All patients were regularly examined, and detailed clinical evaluations were conducted. All patients were followed both clinically and radiologically on the 12th postoperative day for suture removal and at regular intervals at six weeks, three months, six months, and one year.

The benefits of this approach to tibia nailing were assessed based on fluoroscopy exposure, surgical duration, ease of reduction, decreased variability in limb positioning during the procedure (unlike conventional tibia nailing methods), functional outcome, postoperative anterior knee pain, and union rate. All patients were assessed for their functional outcomes based on modified Lysholm knee scores, as shown in Table 1.

**Table 1:** Modified Lysholm score.

Limp	Support	Locking	Instability	Pain	Swelling	Stair climbing	Squatting	Total count
None = 5	None = 5	None = 15	None = 25	None = 25	None = 10	No problem = 10	No problem = 5	Excellent = 91-100
Periodically = 3	Limp = 3	Catching but no locking sensation = 10	Rarely during sports = 20	Slight during exertion = 20	On severe exertion = 6	Slightly impaired = 6	Slightly impaired = 4	Good = 84-90
Severe = 0	Weight-bearing not possible = 0	Occasional locking = 6	Frequently on exertion = 15	Marked during exertion = 15	On slight exertion = 2	Step by step = 2	Up to 90° = 2	Fair = 65-83
		Frequently = 2	Occasionally in daily activity = 10	Marked on walking 2 km or more = 10	Constant = 0	Impossible = 0	Impossible = 0	Poor <=64
		Locked joint = 0	Frequently in daily activity = 5	Marked on walking less than 2 km = 5				
			With every step = 0	Constant = 0				

Inclusion criteria

Patients aged 18 years or more and with proximal one-third and distal one-third closed tibial shaft fractures or open tibia fractures with Gustilo Anderson type I are included in the study.

Exclusion criteria

Patients with open fractures with Gustilo Anderson types II and III, tibia fractures with intra-articular extensions, knee osteoarthritis, nonunion or malunited tibia fractures, or ipsilateral femur shaft fractures are excluded from the study.

## Results

A total of 32 patients were prospectively studied for clinical, radiological, and functional outcomes of suprapatellar nailing as the definitive treatment for tibia shaft fracture. The most common age group was between 40 and 50 years, which encompassed a total of 20 patients (mean of 62.5%). Of these, 27 were male and five were female patients, owing to male preponderance from road traffic accidents as the sole cause of fractures (100%). A total of 30 tibia shaft fractures were closed fractures, and two cases were grade 1 Gustilo Anderson type with a mean of 93.8% and 6.3%, respectively. All patients underwent the same modality of treatment and were followed up at six weeks, three months, six months, and one year.

All 32 patients had a full range of motion at the knee by six weeks postoperative period. Thirty-one patients had signs of the union at the fracture site, with a mean union time of 12.45 months, and one patient failed to come for regular follow-up and had nonunion with implant failure when he presented at our outpatient department at one year. Two patients had persisting anterior knee pain at one year, as shown in Table 2. All patients were assessed for functional outcomes by modified Lysholm score. Our study showed excellent results with a mean modified Lysholm score of 95, as shown in Table 3.

**Table 2:** Incidence of postoperative complications.

	Frequency	Percentage (%)
Anterior knee pain	2	6.3
Non union and implant failure	1	3.1
No complications	29	90.6
Total	32	100

**Table 3:** Functional outcomes based on modified Lysholm score.

	Frequency	Percentage (%)
Excellent	23	71.9
Good	9	28.1
Total	32	100

**Figure 1:**
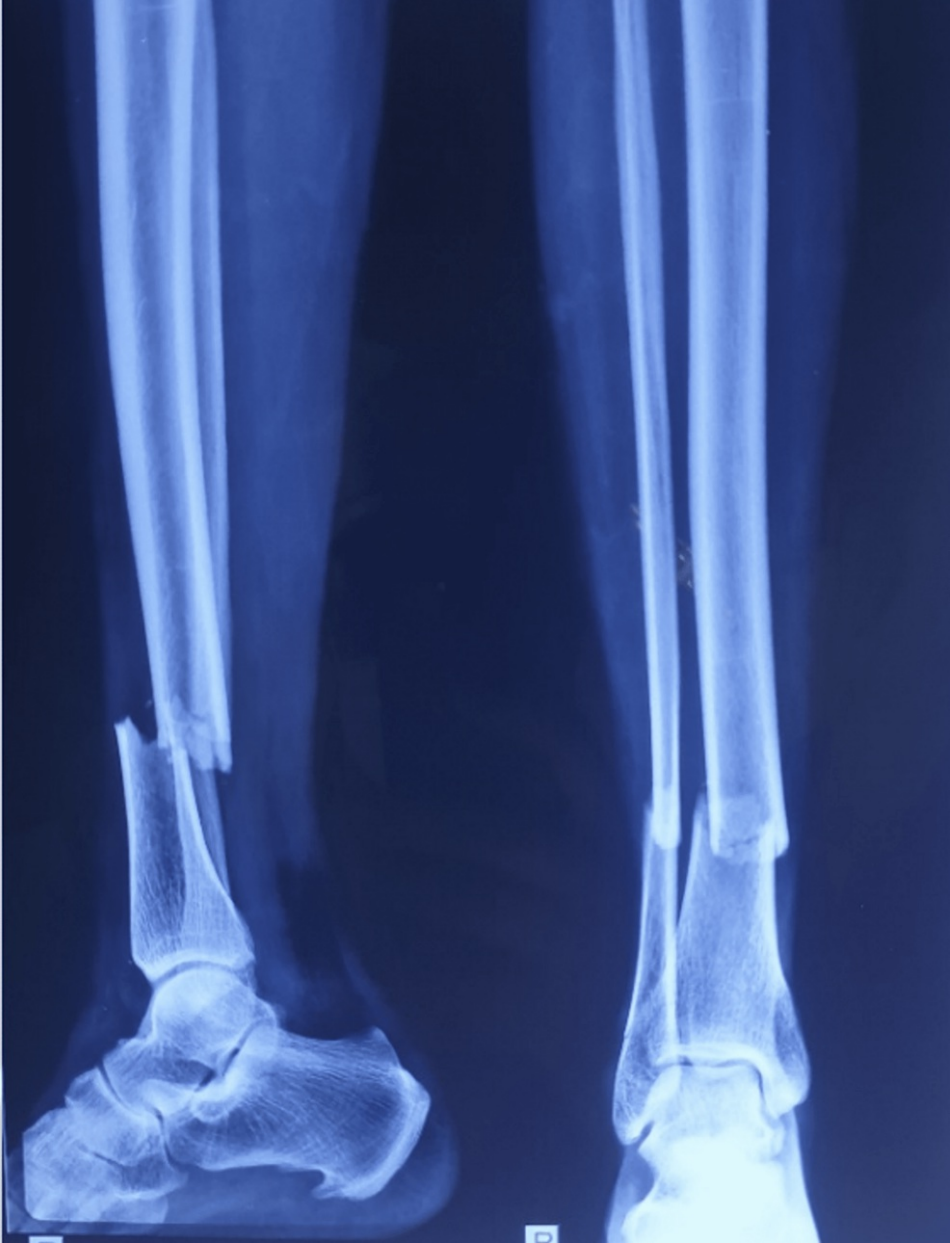
Case illustration: preoperative radiograph.

**Figure 2:**
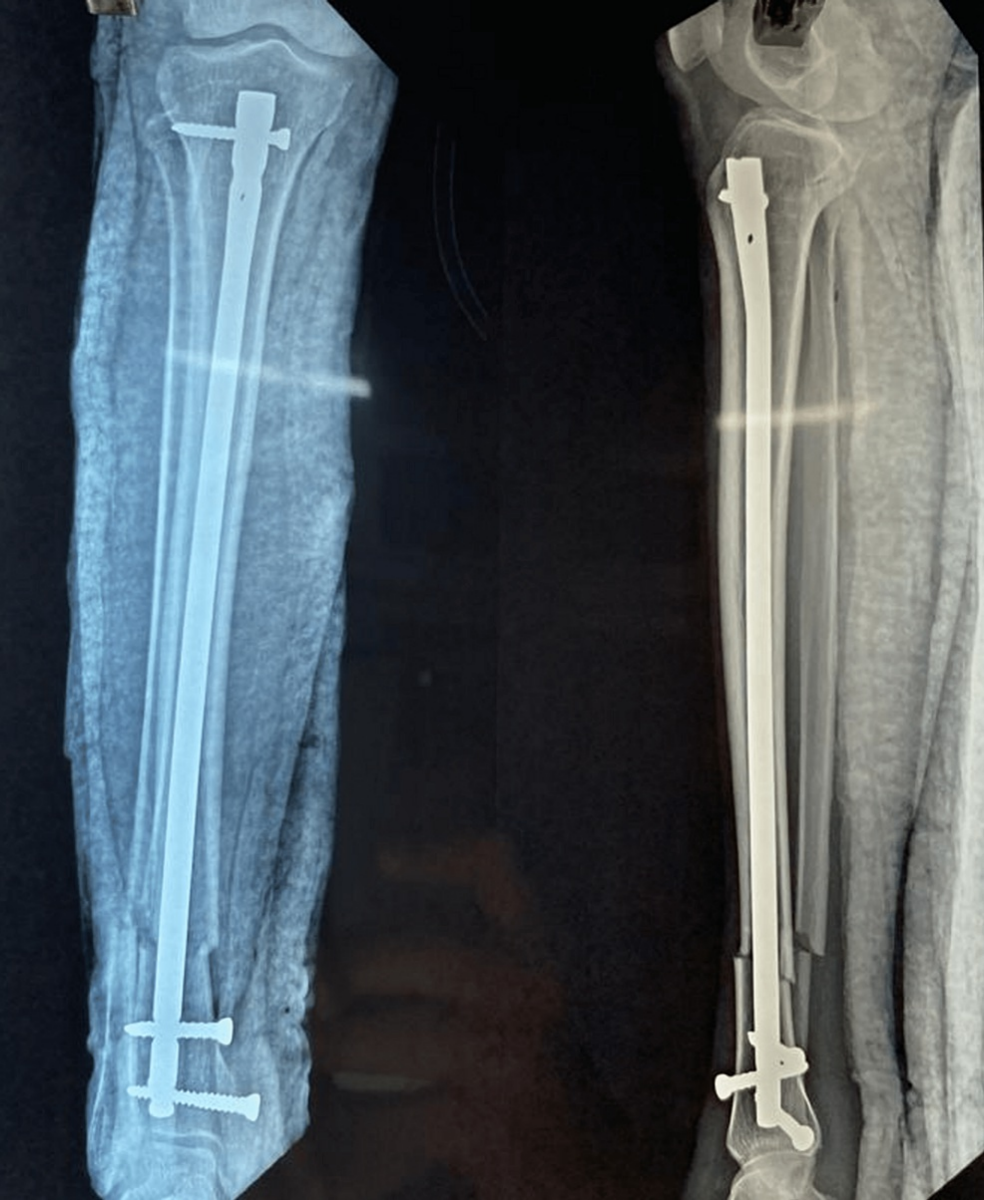
Immediate postoperation.

**Figure 3:**
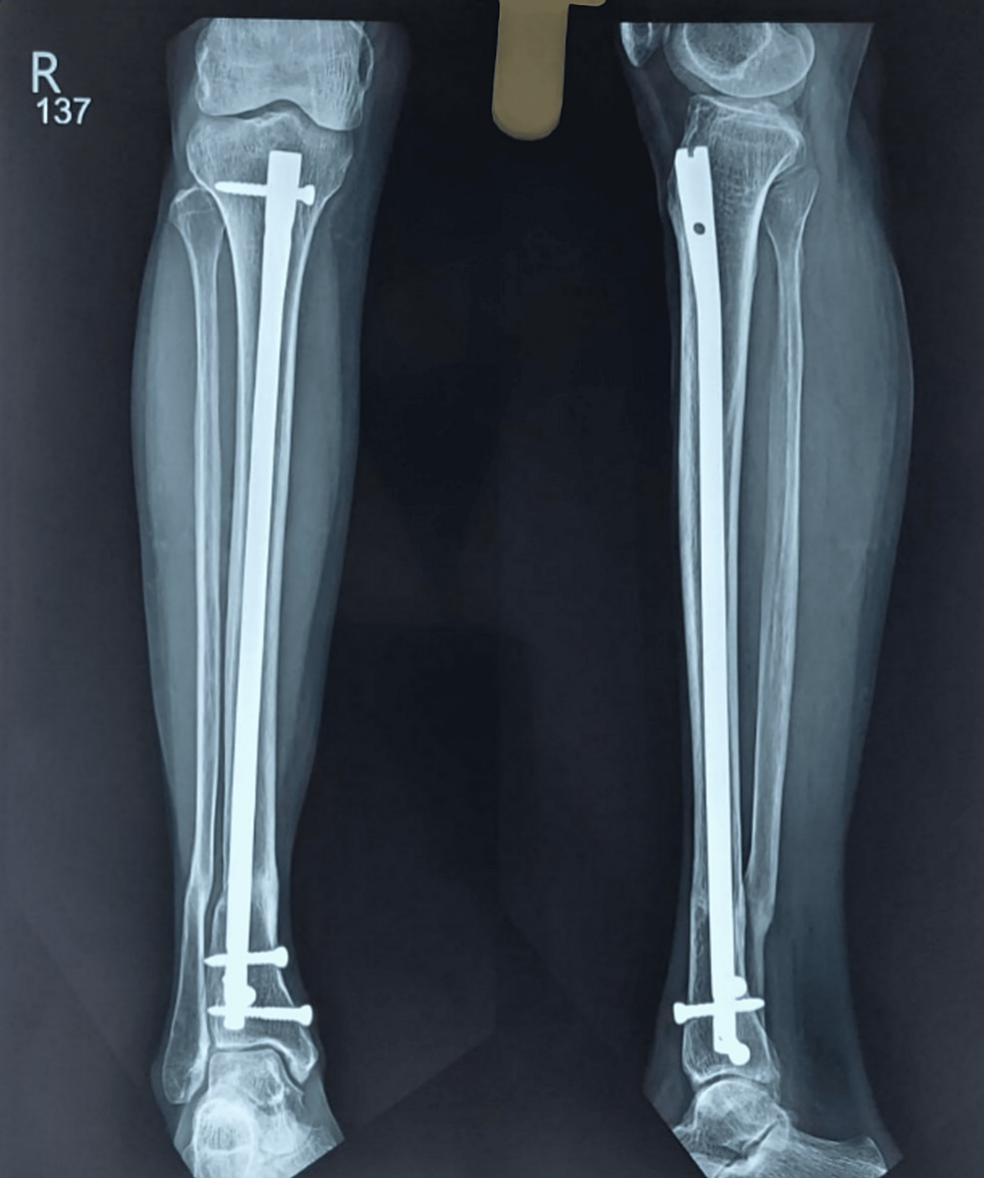
One-year follow-up.

**Figure 4:**
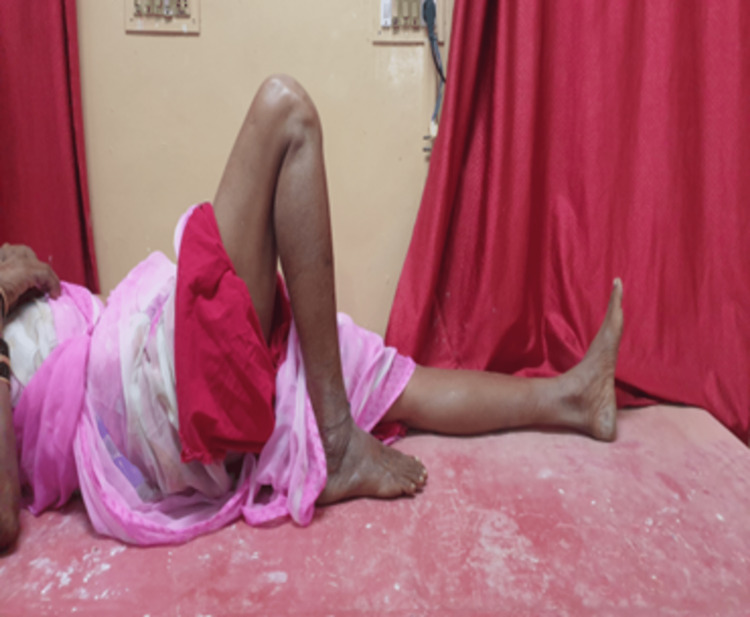
Range of movement - complete knee flexion.

**Figure 5:**
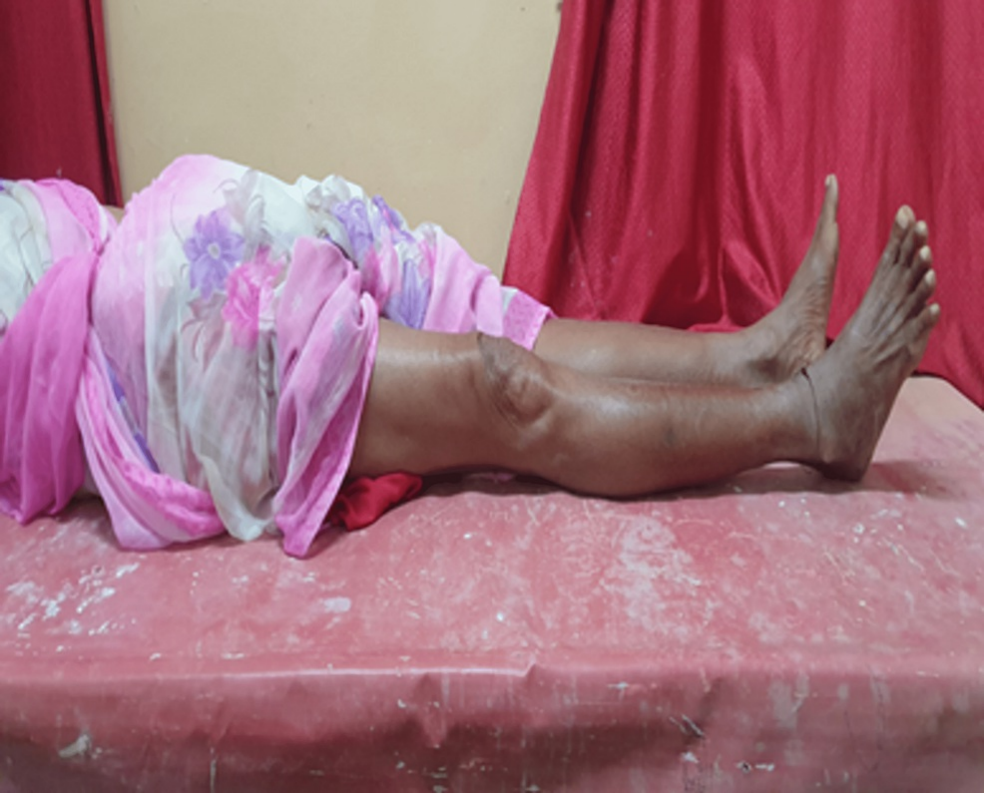
Range of movement - complete knee extension.

Case A case illustration is shown in Figures 1-5. 

## Discussion

The suprapatellar approach has its main advantage in proximal and distal one-third tibia fractures. Orientation of the medullary canal is in line with the entry portal of the suprapatellar approach, reducing the risk of malunion of proximal and distal tibia fractures. The semi-extended knee position makes it easier to reduce the fracture and maintain reduction. The suprapatellar approach of tibia nailing also reduces exposure to fluoroscopy.

The traditional method of infrapatellar nailing by splitting the patellar tendon requires flexion or hyperflexion of the knee. The most common issue faced in proximal tibia fractures is the anterior pull of the proximal fragment by the quadriceps on knee flexion. Procurvatum deformity of the proximal tibia is a commonly encountered complication [12]. With wide metaphysis of the tibia, it is difficult to control the varus or valgus mal reduction of distal tibial fracture through the infrapatellar approach. This also necessitates the additional procedure of the Poller screw technique.

The dilemma in using this approach to tibia nailing lies in its risk of damaging the chondral surface of the patella, the knee, and the anterior horn of the meniscus. The use of a protective sleeve can reduce the risk of these complications. Only two of our operated patients experienced anterior knee pain. However, several studies found no appreciable differences between the suprapatellar and infrapatellar approaches in terms of pain, range of knee motion, or knee functional score [13-15]. In their study, Gaines et al. described lower chances of damage to articular structures with suprapatellar nailing [16].

In our study, we used a protective sleeve for the patellofemoral joint, which further prevented the incidence of chondral damage. The availability of multiple locking options for proximal metaphysis and lesser Herzog bend compared to earlier nails has provided better stability in terms of the design of the implants used in suprapatellar nailing. According to a study by Serbest et al., older nails with acute Herzog bend and lesser locking options have contributed to implant-related failures [17,18]. There has been no evidence of anterior knee pain [19]. Good functional outcomes were found in similar studies based on suprapatellar nailing [20,21].

Technique

**Figure 6:**
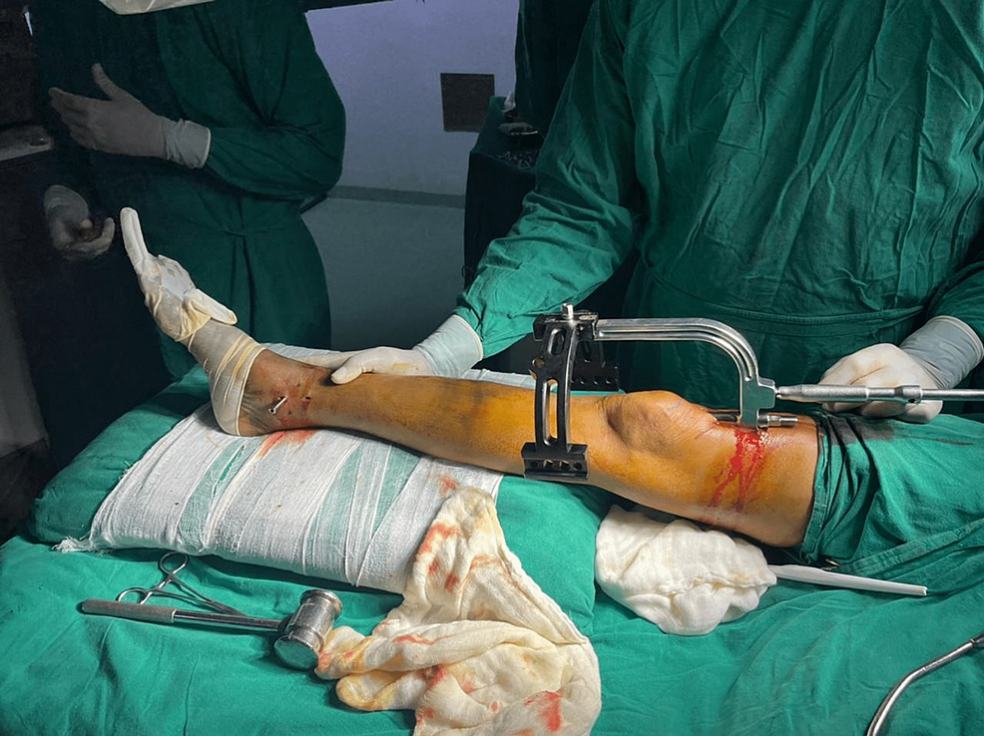
Semi-extended knee position.

**Figure 7:**
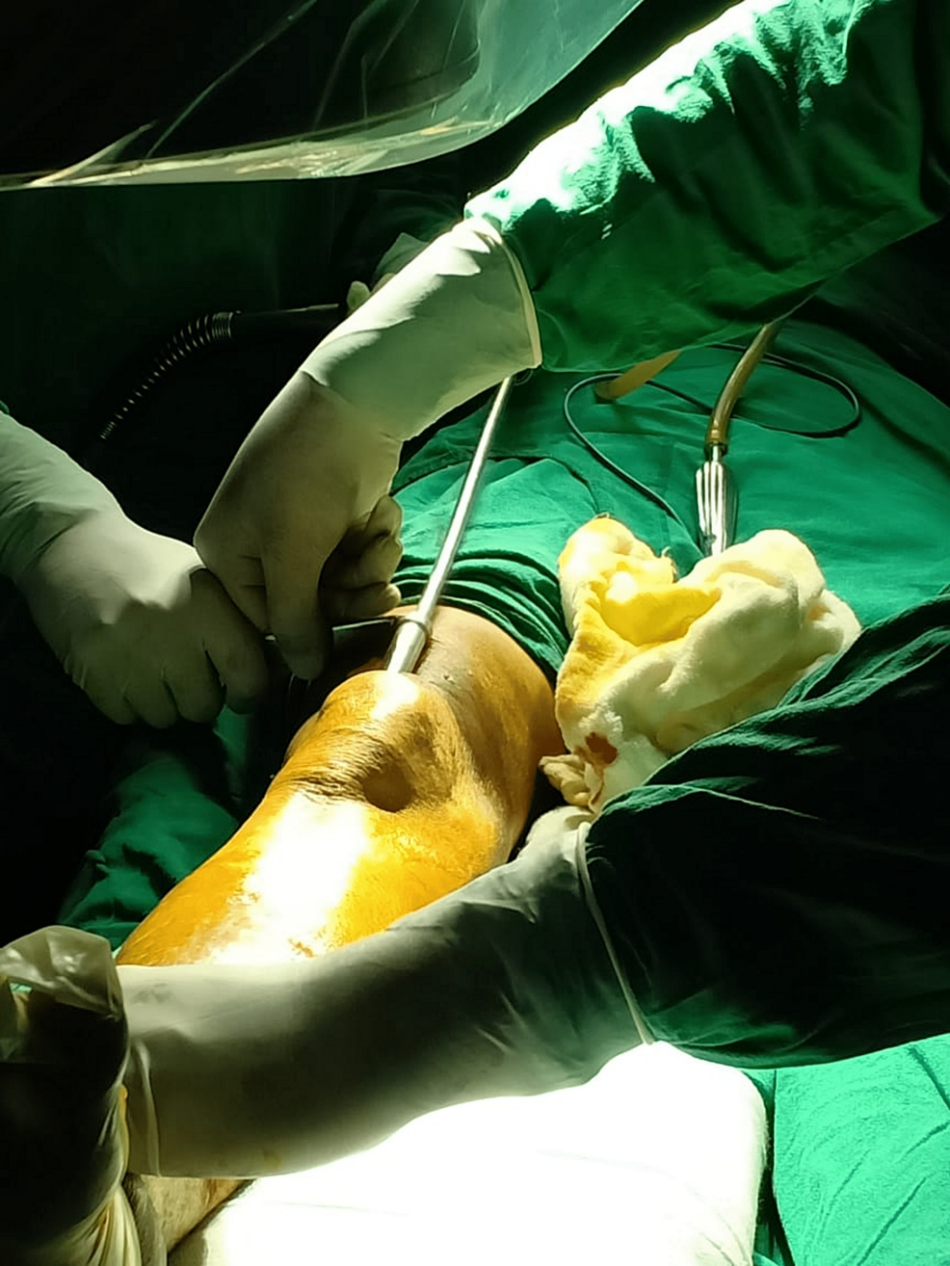
Protective sleeve.

**Figure 8:**
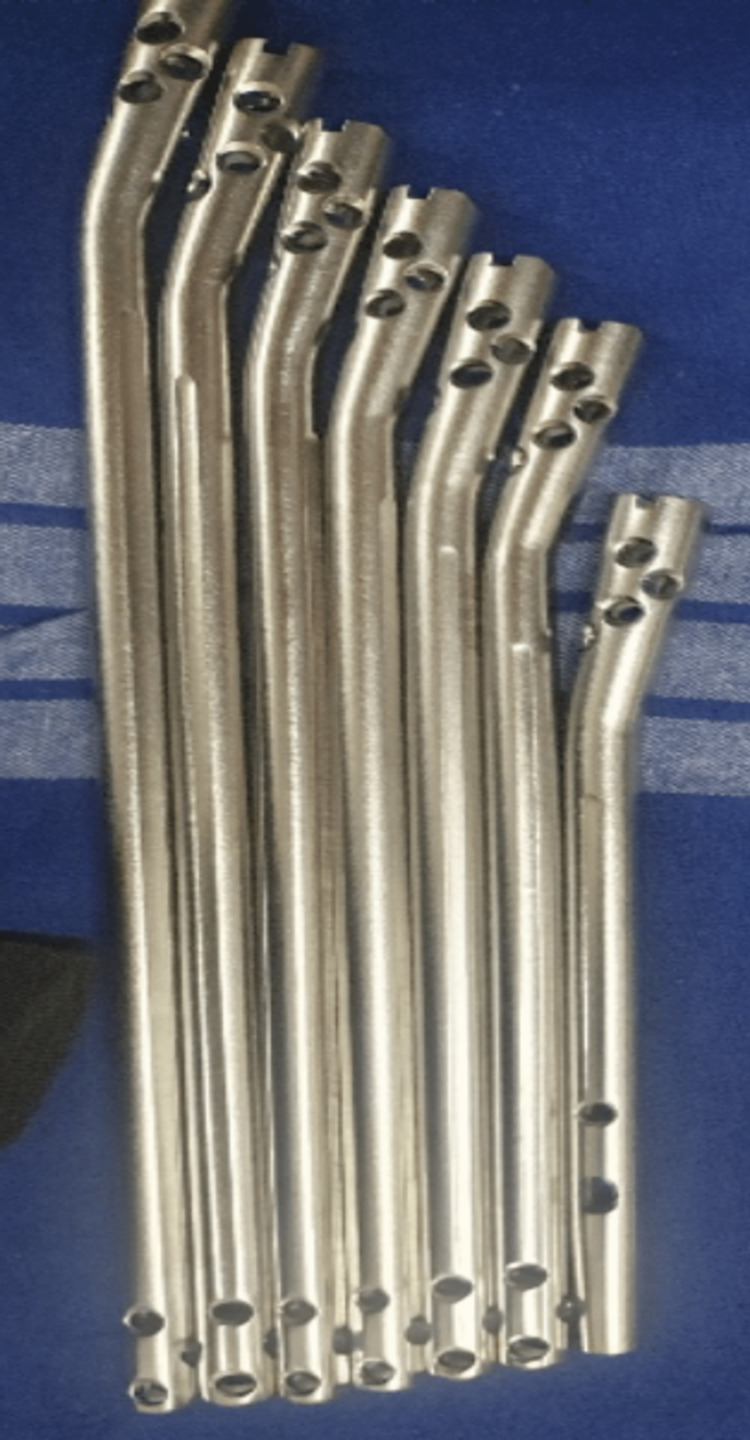
Multiple proximal interlocking options.

The patient is positioned supine with knees semi-extended. Twenty degrees of flexion at the knee was achieved with a bolster, as shown in Figure 6. Skin incisions started 2 cm above the proximal pole of the patella and extended 5 cm proximally, and the quadriceps tendon was visualized. A full-thickness longitudinal incision was made over the substance of the tendon in line with the skin incision. Finger dissection was done to access the patellofemoral joint. If the patella femoral recess is deemed tight, a medial or lateral retinaculum release can be performed. An entry awl passed into the patella femoral space using a protective sleeve, as shown in Figure 7. The entry point is confirmed in both the anteroposterior and lateral views under the image intensifier. The ideal entry point is medial to the lateral tibial spine, 9 mm lateral to the center of the tibial plateau in an anteroposterior view. On the lateral view, the ideal entry is anterior to the articular surface. Reamed suprapatellar tibia nailing was done for fixation of the tibia fracture. Proximal locking was done using a custom jig with multiple locking options, as shown in Figure 8. A distal locking screw was inserted by a freehand technique. A thorough wash was given, and the quadricep tendon was sutured to full thickness, the skin was sutured, and a sterile dressing was applied.

Limitations of the study

A long-term study duration is needed to assess the incidence of patella femoral arthritis.

## Conclusions

The ideal modality of treatment for a closed tibia fracture has been reamed interlocking intramedullary nails with various insertional approaches. Our study describes surgical hints in performing a safe and convenient technique for suprapatellar nailing of tibia fractures. This technique has benefits over the conventional infrapatellar methods in terms of the semi-extended position of the knee, which indirectly facilitates ease of reduction, less fluoroscopy time, and decreased risk of malunion, with far fewer complications of anterior knee pain with excellent functional outcome results based on modified Lysholm knee scores. Further prolonged follow-up studies can validate the potential risk of early arthritis.

Our study concludes that suprapatellar nailing of tibia shaft fractures in a semi-extended knee position is an excellent and innovative method of nailing and prompts further innovations in the field of orthopedics.

## References

[1] Court-Brown CM, Caesar B (2006). Epidemiology of adult fractures: a review. Injury.

[2] Larsen P, Lund H, Laessoe U, Graven-Nielsen T, Rasmussen S (2014). Restrictions in quality of life after intramedullary nailing of tibial shaft fracture: a retrospective follow-up study of 223 cases. J Orthop Trauma.

[3] Larsen P, Elsoe R, Hansen SH, Graven-Nielsen T, Laessoe U, Rasmussen S (2015). Incidence and epidemiology of tibial shaft fractures. Injury.

[4] Anandasivam NS, Russo GS, Swallow MS (2017). Tibial shaft fracture: a large-scale study defining the injured population and associated injuries. J Clin Orthop Trauma.

[5] Böhler L (1948). Medullary Nailing of Küntscher, rev. edn. Rev.

[6] Cazzato G, Saccomanno MF, Noia G, Masci G, Peruzzi M, Marinangeli M, Maccauro G (2018). Intramedullary nailing of tibial shaft fractures in the semi-extended position using a suprapatellar approach: a retrospective case series. Injury.

[7] Tornetta III P, Riina J, Geller J, Purban W (1999). Intraarticular anatomic risks of tibial nailing. J Orthop Trauma.

[8] Cole JD (2006). Distal tibia fracture: opinion: intramedullary nailing. J Orthop Trauma.

[9] Sanders DW, Bhandari M, Guyatt G (2014). Critical-sized defect in the tibia: is it critical? Results from the SPRINT trial. J Orthop Trauma.

[10] Wang Y, Chen L, She R, Dai T, Zhang Y, Lan J, Wu S (2019). Efficacy of suprapatellar versus infrapatellar approach in tibial intramedullary nail fixation for tibial fracture: a systematic review and meta-analysis. Chinese J Trauma.

[11] Franke J, Homeier A, Metz L (2018). Infrapatellar vs. suprapatellar approach to obtain an optimal insertion angle for intramedullary nailing of tibial fractures. Eur J Trauma Emerg Surg.

[12] Freedman EL, Johnson EE (1995). Radiographic analysis of tibial fracture malalignment following intramedullary nailing. Clinical Orthop Rel Res.

[13] Jones M, Parry M, Whitehouse M, Mitchell S (2014). Radiologic outcome and patient-reported function after intramedullary nailing: a comparison of the retropatellar and infrapatellar approach. J Orthop Trauma.

[14] Chan DS, Serrano-Riera R, Griffing R (2016). Suprapatellar versus infrapatellar tibial nail insertion: a prospective randomized control pilot study. J Orthop Trauma.

[15] Courtney PM, Boniello A, Donegan D, Ahn J, Mehta S (2015). Functional knee outcomes in infrapatellar and suprapatellar tibial nailing: does approach matter?. Age.

[16] Gaines RJ, Rockwood J, Garland J, Ellingson C, Demaio M (2013). Comparison of insertional trauma between suprapatellar and infrapatellar portals for tibial nailing. Orthopedics.

[17] Wolinsky PR, Dennis D, Crist BD, Curtiss S, Hazelwood SJ (2011). The biomechanics of varied proximal locking screw configurations in a synthetic model of proximal third tibial fracture fixation. J Orthop Trauma.

[18] Buehler KC, Green J, Woll TS, Duwelius PJ (1997). A technique for intramedullary nailing of proximal third tibia fractures. J Orthop Trauma.

[19] Serbest S, Tiftikçi U, Çoban M, Çirpar M, Dağlar B (2019). Knee pain and functional scores after intramedullary nailing of tibial shaft fractures using a suprapatellar approach. J Orthop Trauma.

[20] Fu B (2016). Locked META intramedullary nailing fixation for tibial fractures via a suprapatellar approach. Indian J Orthop.

[21] Sun Q, Nie X, Gong J, Wu J, Li R, Ge W, Cai M (2016). The outcome comparison of the suprapatellar approach and infrapatellar approach for tibia intramedullary nailing. Int Orthop.

